# Characterization of mechanical strain induced by lead-bismuth eutectic (LBE) freezing in stainless steel cup

**DOI:** 10.1016/j.heliyon.2020.e03429

**Published:** 2020-02-21

**Authors:** Naoya Odaira, Shigeru Saito

**Affiliations:** a University of Fukui, Kanawa-cho 1-3-33, Tsuruga, Fukui, 914-0054, Japan; b Japan Atomic Energy Agency, Shirakata 2-4, Tokai, Ibaraki-pref, 319-1106, Japan

**Keywords:** Materials science, Materials chemistry, Freeze sealed valve, Metallographic observation, LBE expansion

## Abstract

Lead-bismuth eutectic (LBE) is a candidate liquid metal coolant for a fast reactor, especially accelerator driven system (ADS). Freeze sealed valve is a candidate design to be possible to add passive safety to the reactor. On the other hand, since LBE is known that it causes expansion after its solidification, quantitative evaluation of the stress to the pipe produced by the LBE expansion should be considered. Many researchers produced related data for the expansion, however, evaluations of the strain by LBE expansion was barely reported. Therefore, the strain measurement using a stainless steel cup and the stress evaluation was performed together with visual observation using an optical microscopy. The results indicated keeping above room temperature (RT) was a significantly effective way to reduce the strain to the pipe.

## 1 Introduction

**Figure 1:**
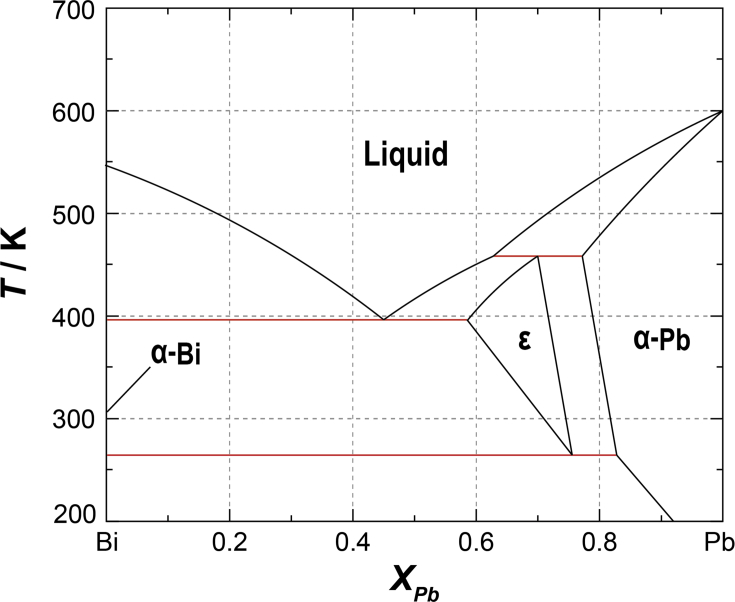
Pb–Bi system [6].

Lead-bismuth eutectic (LBE) is a promising candidate liquid metal coolant for a fast reactor, especially for accelerator driven system (ADS) [1]. LBE has significant advantages in fast reactor. For example, LBE doesn't react with air violently in case of a loss of coolant accident unlike sodium which is another candidate of liquid metal coolant in fast reactor. On the other hand, LBE has a specific property in its density. It is known that LBE gradually expands itself with time after its solidification. This phenomenon is known that is not like ice which expansion occurs during solidification, which is not “after” solidification like LBE. Glasbrenner [2] reported LBE had been continued expansion more than one year and Takeda reported similar dependence with time for one month [3]. Thus, short term of solidifying LBE probably is less problem. However, if longer term of solidifying LBE were required, LBE expansion is possible to produce stress to pipes. According to Zucchini [4], LBE expansion is provided by reaching its equilibrium. During liquid-solid phase transition, crystal grains are separated into two parts (L→S + S) called α-Bi and ε (intermediate phase of Pb and Bi). Okada [5] confirmed the transition from ε to α-Bi by using neutron diffraction. The ratio of α-Bi was estimated as 25 % at melting point from phase diagram [6] (see Figure 1). On the other hand, the ratio increase to 32 % at room temperature (RT) by transforming ε to α-Bi. Increasing α-Bi causes LBE expansion since α-Bi (9.780 gcm^−3^ at 20 °C) [7] has lower density than ε (11.17 gcm^−3^) [8]. Thus, this phenomenon is related to diffusion of elements and strongly depends on heat treatment obviously.

By freezing LBE in piping, we can create a freeze sealed valve wherever we want, if continuous cooling were provided. Especially in experimental fast reactor or its related facility, freeze sealed valve at the pipe located between drain tank and primary coolant system may prevent public exposure passively since LBE highly contains radioactive nuclides. LBE in the primary loop would be over 400 °C during operation, thus cooling of the part should be performed to keep it solid. In case of an accident such as station black out, freeze sealed valve is going to be melted quickly because of loss of continuous cooling. As a result of abovementioned events, LBE in primary system may be drained into the tank without any operation.

Although LBE compressive properties were revealed by Dai [9], strain measurement was barely reported. Therefore, strain measurement to stainless steel cup produced by LBE expansion was investigated together with metallographic observation in this study.

## 2 Experiment

**Figure 2:**
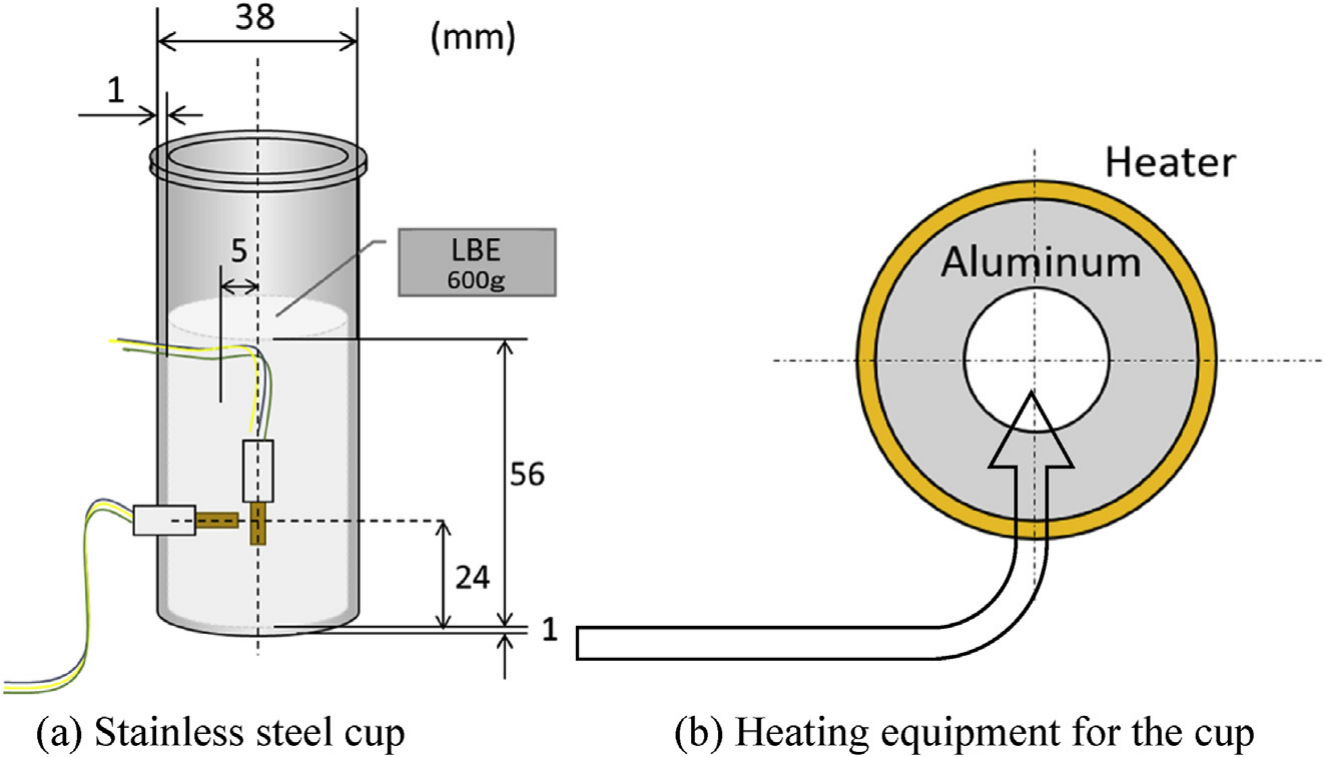
Schematic view of (a) stainless steel cup filled by LBE with sticking strain gauge and (b) heating equipment for the cup.

Stainless steel cup (φ = 36 mm, l = 90 mm, type 304 stainless steel) was prepared to measure strain (see Figure 2) LBE alloy supplied by Senju Metal Industry (Japan) was used for the experiments in this study. First, 600 g of LBE ingots was put into the cup and it was heated up to 150 °C in the electric furnace under flowing inert gas (${\text{N}}_{2}$) and maintained until melting all ingots. Floated oxidized layer was removed to be clean the surface. Then, the cup was cooled with different cooling rate (max. cooling rate was approximately 1.4 K/min). Cooling was controlled by a heater and a fan located at the bottom of the furnace. After reaching RT (25 °C), strain gauges were stuck for measuring hoop and axial strain on the cup at 24 mm from the bottom and the measurement started. The location to stick the strain gauge was decided based on pre-calculation result performed by the calculation by finite element method with using artificial thermal expansion instead of LBE expansion. In addition to one-point measurement, four points measurement including three points for hoop strain (17, 34, and 51 mm from the bottom) and one point for axial strain (24 mm from bottom) carried out. Temperature compensated strain gauges (1.4 mm×5 mm grid size, ±1% uncertainty in the gauge factor at 20 °C) supplied by Kyowa Electronic Instruments Co. were used in the experiment. For precise strain measurement, the cup was put in a constant temperature oven over the measurement. Sampling rate was 1 point/min. The measured time was changed with experimental condition since LBE expansion behavior depends on heat treatment.

Metallographic examination was performed for LBE specimens cooled with three different cooling rates (1/8, 1/2, and 1.4 K/min) by using optical microscopy (ECLIPSE MA200 produced by Nikon). Grain size was evaluated by image processing software (NIS-ELEMENTS produced by Nikon). Minimum recognition size (i.e. pixel size) was 0.034 μm. LBE specimens were polished by using SiC emery papers, diamond pastes and SiO_2_ suspension. Each specimen was prepared by different experiment with using small pieces of LBE ingot to perform the same cooling rate as the strain measurement used.

## 3 Results and discussion

### 3.1 Strain measurement

**Figure 3:**
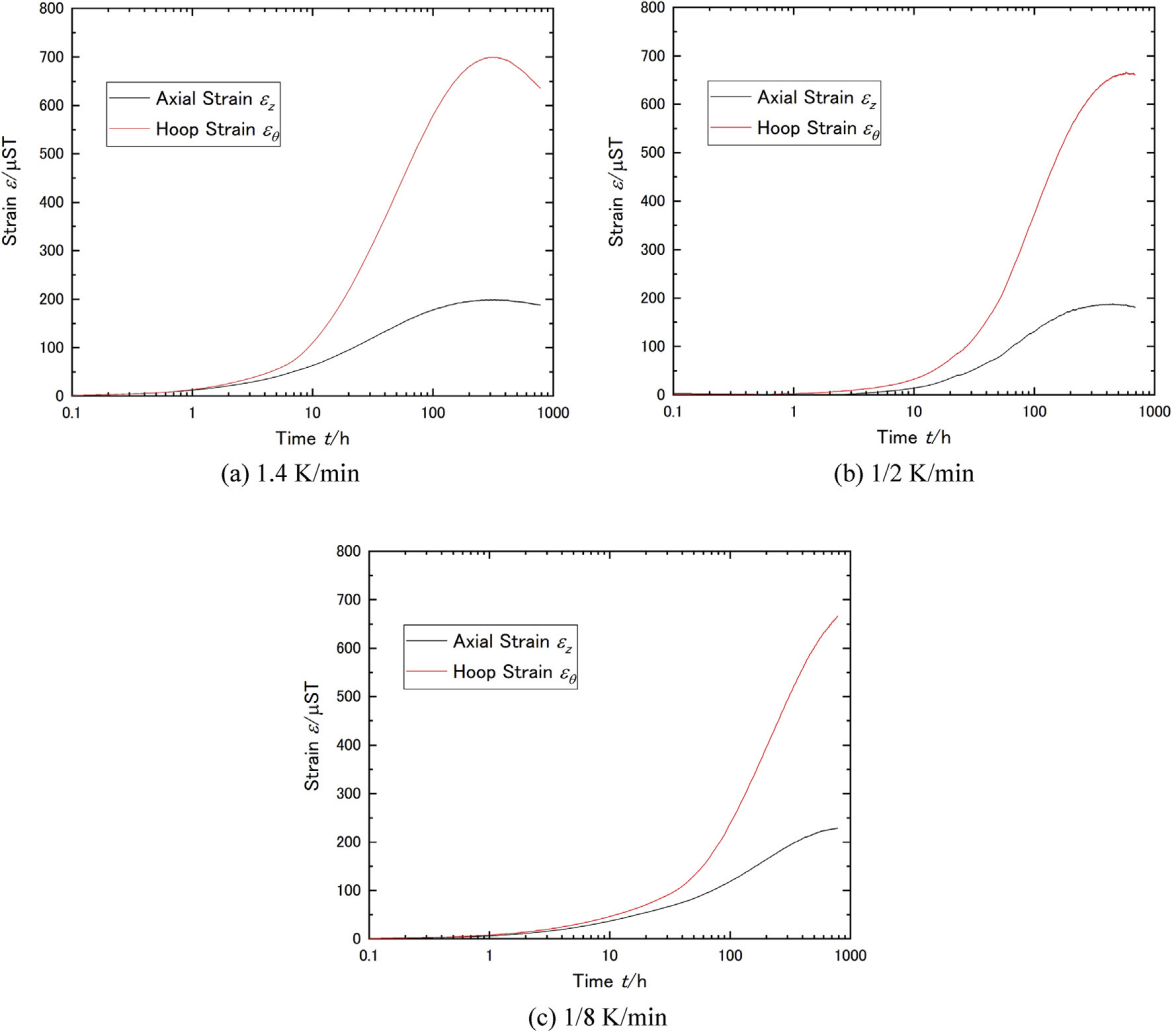
Measured strain with different cooling rate ((a) 1.4K/min, (b) 1/2 K/min and (c) 1/8 K/min).

**Figure 4:**
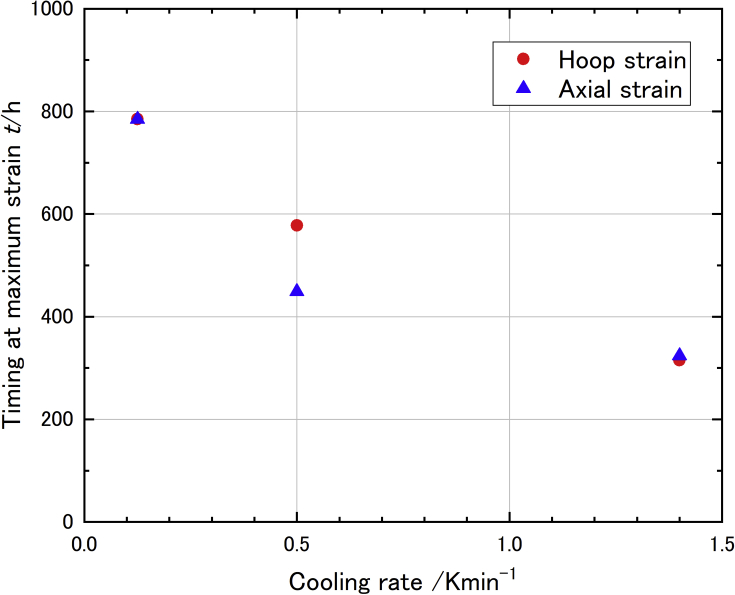
Maximum reaching time with cooling rate.

Figures 3(a) to (c) show measured strain with different cooling rate (1/8, 1/2, and 1.4 K/min). The relation between strain and time which were logarithmically linear in each condition which might be related to LBE expansion. Maximum strain points were observed in each experiment, thus, the maximum strain and the time to reach maximum strain were the main results in this paper. In the 1/8 K/min case, although we couldn't observe maximum reached strain, it was treated as maximum strain since it looks close to reach maximum from its logarithmic curve. At first, we had expected the maximum strain has strong relation with cooling rate, however, only small difference was observed in the maximum strain (summarized in Table 1). Although the cooling rate dependency was not observed in this experiment, the time to reach the maximum had clear dependency which decreasing the time with increasing cooling rate (see Figure 4).

**Table 1:** Evaluated strain and stress produced by LBE.

Cooling rate (K/min)	Max. hoop strain ($\times 10^{-6}$)	Max. hoop stress (MPa)	Max. axial strain ($\times 10^{-6}$)	Max. axial stress (MPa)	Internal pressure by LBE (MPa)
1.4	700.0	186.0	199.0	118.4	10.3
1/2	666.5	176.9	187.6	112.3	10.1
1/8	666.9	181.3	229.3	122.2	10.1

**Figure 5:**
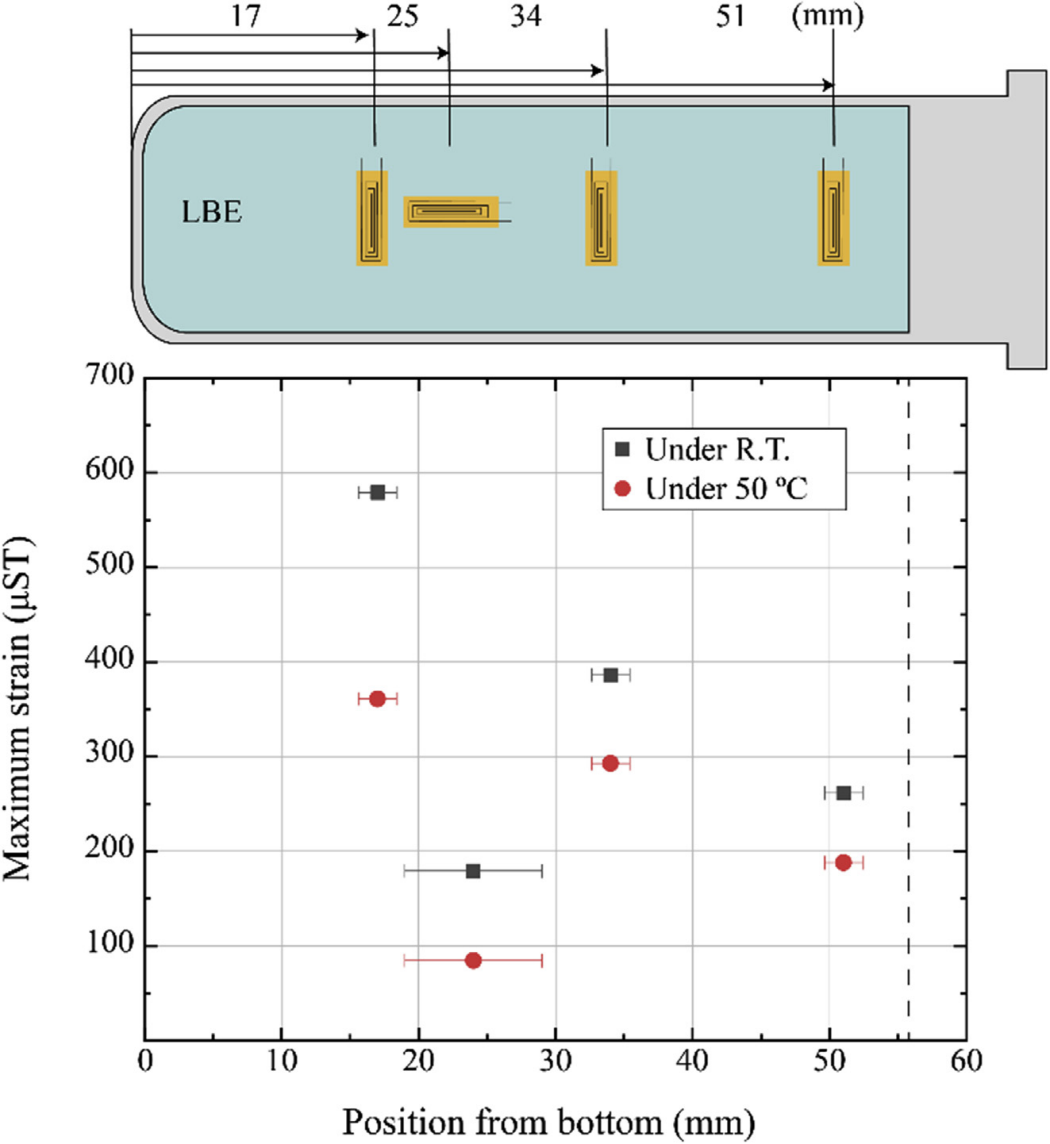
Comparison of measured strain under 50 °C and 25 °C with position from bottom. Error bar showed grid size of strain gauge.

In addition to one-point measurement, two kind of four points measurement at RT and 50 °C were performed (17, 34, and 51 mm for hoop strain and 24 mm for axial strain). Comparison between the maximum strains at RT and those at 50 °C showed up to approximately 40 % reduction of strain was observed for all measured positions (see Figure 5).

**Figure 6:**
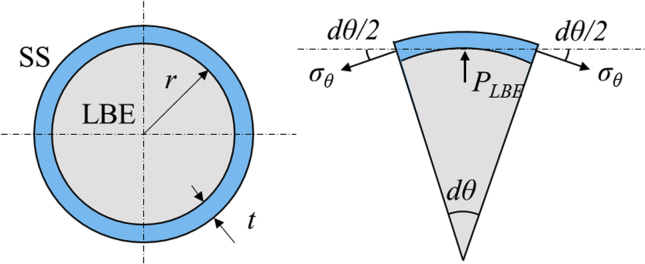
Simplified model for the stainless steel cup with LBE expansion.

Since hoop and radial strain of the stainless-steel cup should be the same, it was considered that hoop strain of the cup could be the same value as the amount of LBE linear expansion at first. However, comparison of the measured strain with the LBE linear expansion taken from Takeda [3] revealed enormous discrepancy with our results which the expansion was revealed approximately $\Delta V/V=3000\times 10^{-6}$ at 30 h after solidification by Takeda, on the other hand, present results showed maximum 700μST of the strain (see Table 1). This indicated the phenomenon was not only related to the LBE expansion but also related to the LBE deformation inside of the cup. Assuming, thin cylinder approximation (see Figure 6) was applied to estimate internal stress produced by LBE with following equations:

(1) $$P_{LBE}=\frac{t}{r}\sigma_{\theta }$$

(2) $$\sigma_{\theta }=\frac{E}{1-\upsilon^{2}}\left(\varepsilon_{\theta }+\upsilon \varepsilon_{z}\right)$$

where $P_{LBE}$ is internal pressure induced by LBE expansion, *t* is thickness (= 1 mm), *r* is radius of the cup (= 18 mm), $\sigma_{\theta }$ is hoop stress calculated from measured strain, *E* is Young's modulus (= 193 GPa), υ is Poisson's ratio (= 0.43), and $\varepsilon_{\theta }$ and $\varepsilon_{z}$ are the measured hoop and axial strain, respectively.

Axial stress was able to be calculated as well from hoop and axial strain by following equation. The result was summarized in Table 1 together with hoop stress.

(3) $$\sigma_{z}=\frac{E}{1-\upsilon^{2}}\left(\varepsilon_{z}+\upsilon \varepsilon_{\theta }\right)$$

It should be mentioned that the calculated axial stress was relatively large compared with capsule approximation which was derived from following equation although the calculated axial stress $\sigma_{z}$ was less problem than hoop stress $\sigma_{\theta }$.

(4) $$\sigma_{z}=\frac{P_{in}}{2t}$$

$P_{in}$ indicates internal pressure equally applied in the capsule by gas or liquid generally. In case of this approximation, axial stress is equal to half of hoop stress. On the other hand, the result in this experiment showed the axial stress was larger than the half of hoop stress, the ratio was approximately 0.65. This might be related to the adhesion between LBE and stainless steel, however, this was not clearly revealed.

Thin cylinder approximation was often applied to pipes contained pressurized liquid and gas which were stress distribution doesn't exist inside of the pipes. On the other hand, unfortunately, since the cup used in the present experiment was filled by solid LBE which was heterogeneously stressed and it should have been deformed. Although stress distribution inside of the cup was not revealed in this study, above mentioned $P_{LBE}$ should have been agreed at the boundary between LBE and the cup, at least. So that maximum $P_{LBE}$ was considered as compressive yield stress of LBE. Comparison of $P_{LBE}$ and compressive yield stress taken from Dai [9] agreed well. Calculated internal pressure induced by LBE summarized in Table 1 showed the internal pressure was approximately 10 MPa. The order of the strain rate in this experiment might be $10^{-8}$ 1/s or less although the strain rate was not determined since it should be equal to the expansion rate which varied with time dynamically. The compressive yield stress of LBE taken from Dai [9] indicated that it was 12.1 MPa at 20 °C with $1.1\times 10^{-6}\;$1/s of strain rate which was agreed well with the calculated value.

Keeping below melting temperature was proven as a considerably effective way to reduce the strain. Although quantitative evidence was not gained, we believe that the yield compression stress of LBE is a key to resolve it since LBE should have been compressed by stainless-steel and LBE produced tensile deformation of stainless-steel. The compressive yield stress of LBE by Dai [9] showed the compressive yield stress at 52 °C was approximately 0.6 times the yield stress at 20 °C.

### 3.2 Metallographic observation

**Figure 7:**
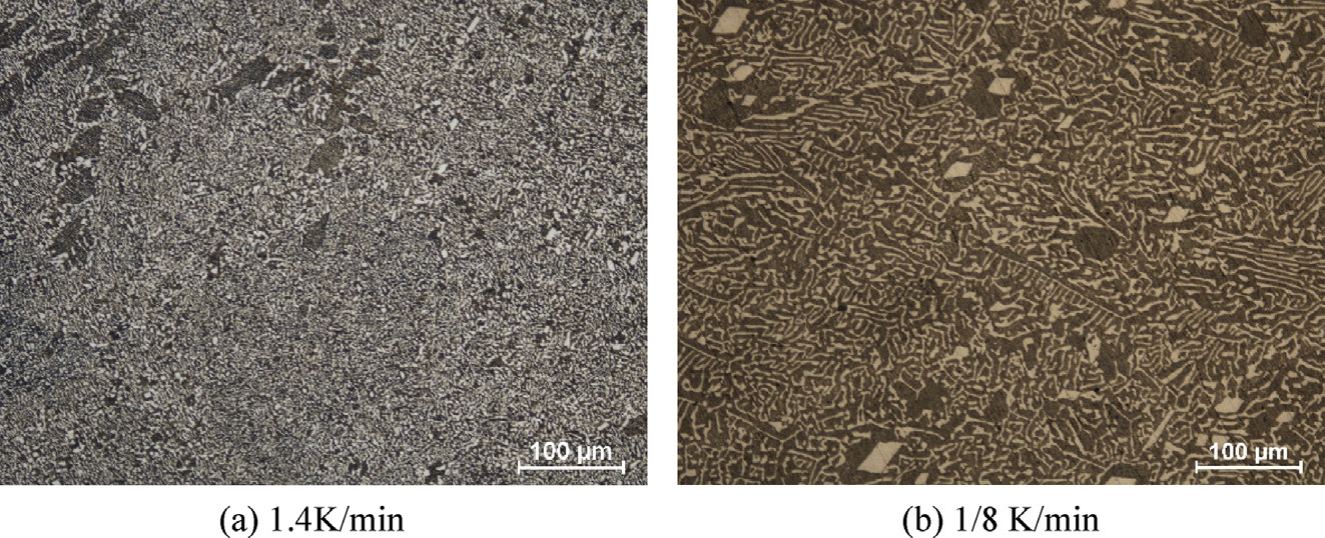
Microstructure overview of LBE with different cooling rate ((a) 1.4 K/min and (b) 1/8 K/min. Bright and dark area indicates α-Bi and ε phase respectively.

Metallographic observation was carried out for LBE specimens with three different cooling rates (1/8, 1/2, and 1.4 K/min). Characteristic eutectic structure was observed in all specimens as indicated in the phase diagram (see Figure 1). Bright and dark regions corresponded to α-Bi and ε phase (${\text{Pb}}_{0.7}{\text{Bi}}_{0.3}$), respectively [2, 5]. The ratio of bright and dark area was approximately 7:3 which agreed well with the phase ratio of α-Bi and ε phase in the phase diagram as in Figure 1. However, the ratio varied with threshold of the contrast and the recognition size. Thus, we couldn't specify the phase ratio from the result of metallographic observation. It was clearly shown that the grain size strongly depended on cooling rate which lower cooling rate causes larger grain size (see Figure 7).

**Figure 8:**
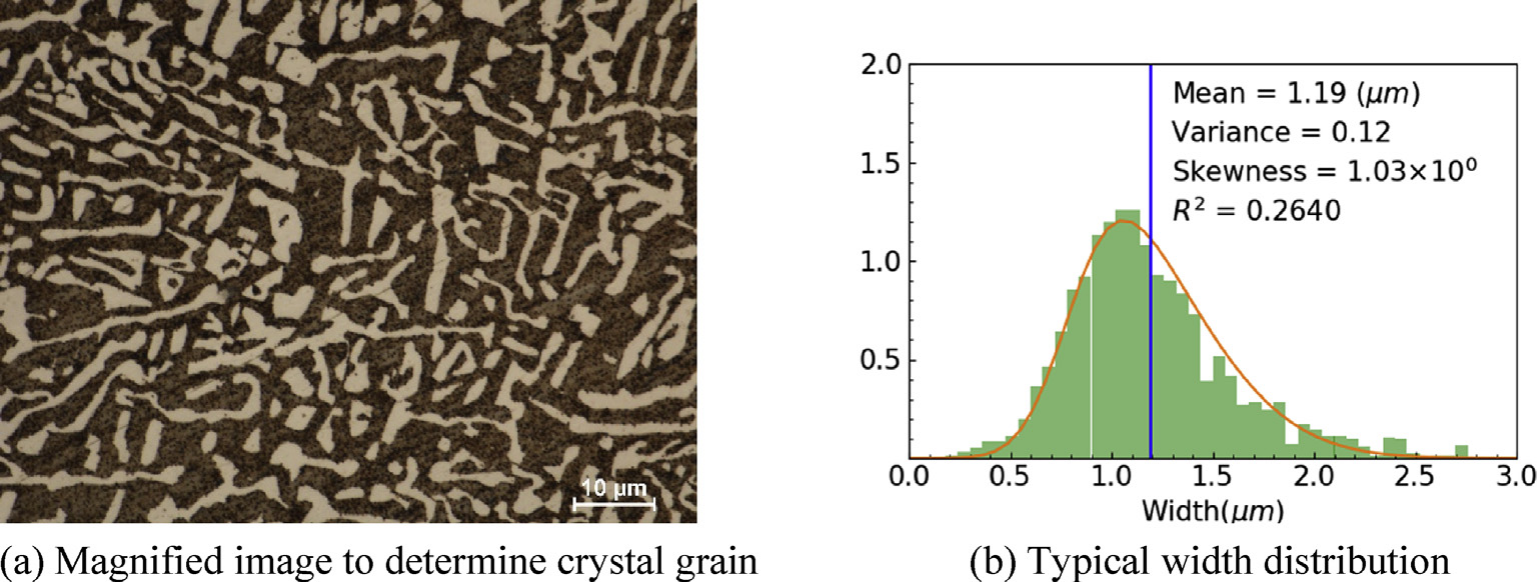
Magnified microstructure and its width distribution. Evaluated width data from image analysis was fitted to skew-normal distribution. (a) Typical magnified image to determine crystal grain width (cooled with 1/2 K/min). (b) Typical grain width distribution for cooled with 1/2 K/min.

**Figure 9:**
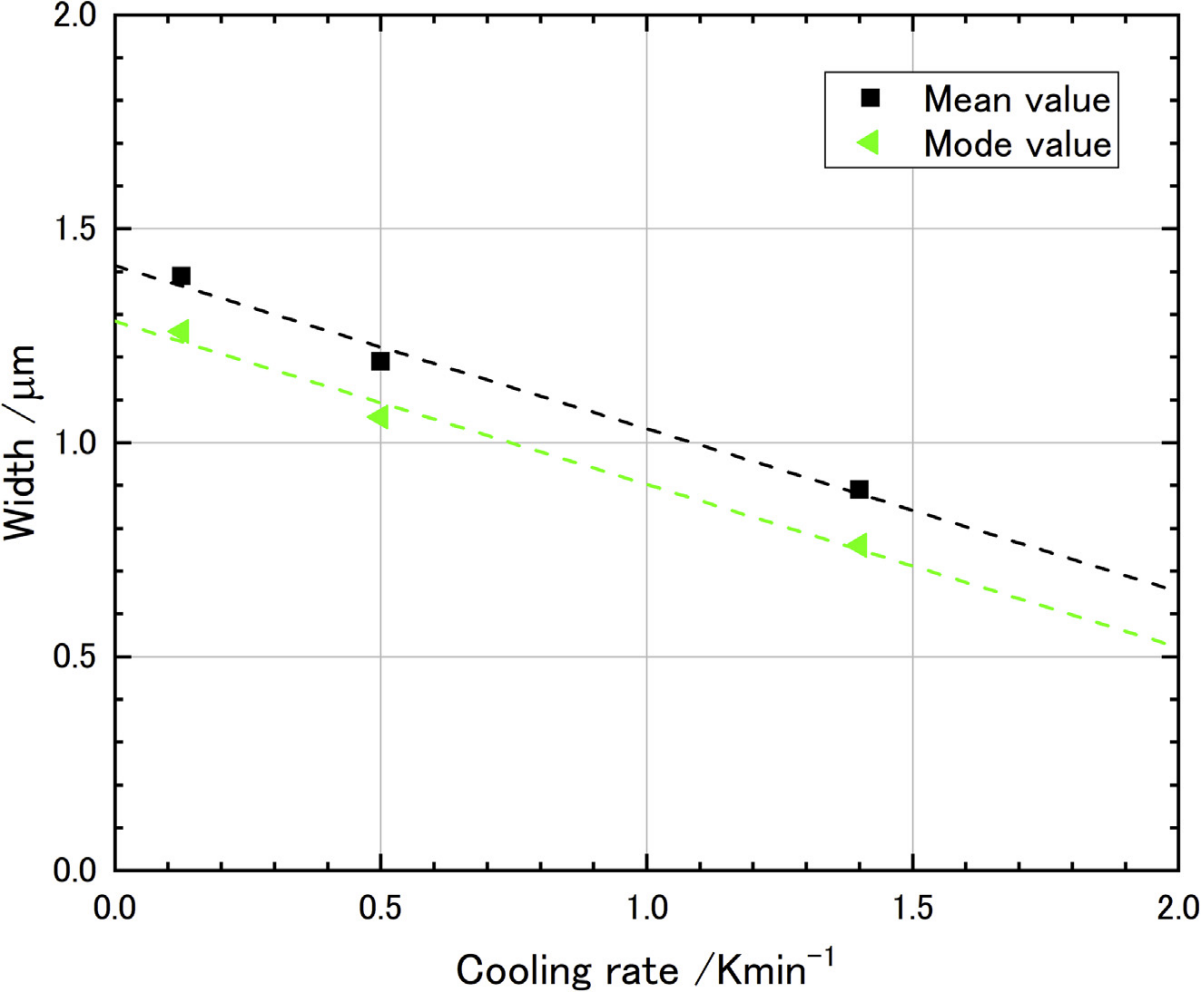
Obtained linear correlation of grain size with cooling rate for α-Bi phase in LBE microstructure.

By using an image processing software, more than 1,000 of areas of α-Bi phase approximated to rectangles from the contrast of the images where randomly selected 15 positions, and then expected width and length were calculated as grain size. Resolution of the software was 0.034 μm which was the pixel size of pictures. Recognized α-Bi area was chosen as a weight of distribution to control domination of small grains. Obtained grain width and length were fitted to the skew-normal distribution [10] and those expected values were calculated. Typical distribution and an image used in the image processing was shown in Figure 8. Obtained data was summarized in Table 2. As a result of the size evaluation, we obtained linear correlation between grain size and cooling rate (see Figure 9).

**Table 2:** Evaluated α-Bi phase grain size distribution with different cooling rate.

Cooling rate (K/min)	Expected grain width (μm)	Variance	Skewness	$R^{2}$
1.4	0.89	0.07	1.4113	0.4889
1/2	1.19	0.12	1.03	0.2640
1/8	1.39	0.22	0.4954	0.5870
Cooling rate (K/min)	Expected grain length (μm)	Variance	Skewness	$R^{2}$
1.4	6.56	31.45	$1.20\times 10^{-4}$	0.006
1/2	7.32	56.94	$1.25\times 10^{-4}$	0.005
1/8	8.81	90.50	$1.32\times 10^{-4}$	0.003

## 4 Discussion

As mentioned above section, the maximum strain points were observed in each experiment. The reason why the maximum strain appeared was still insufficiently revealed since compressive deformation of LBE was a key and the yield stress was difficult to determine. Compressive yield stress was known as temperature and strain rate dependent. Strain rate in this experiment should be equal to the expansion rate which was not determined in the present experiment and not well known until now since it logarithmically depends on time and heat treatment.

The result of strain measurement indicates cooling rate didn't affect to maximum value of the strain whereas the results also indicates cooling rate affect to the time to reach maximum strain. Hence, cooling rate may affect the expansion rate. On the other hand, metallographic observation revealed a relationship between grain size and cooling rate. Therefore, expansion rate and grain size may have a relation.

Grain growth is one of the common phenomena in metal microstructure. Since the transformation of ε phase to α-Bi may be similar phenomenon with grain growth, the relation between grain growth and grain size was investigated. The relation between grain growth rate and grain size is indicated as following:

(5) $$\frac{dD_{g}}{dt}=\frac{K_{0}}{D_{g}}$$

here, $D_{g}$ is grain diameter and $K_{0}$ is velocity parameter. This formula indicates grain growth rate inversely proportional to grain size. In the present study, LBE expansion rate may have inversely proportional to grain size as well as the above formula.

We considered the grain size was not sufficiently suitable to compare with the expansion rate, because the observed α-Bi phase grain was not like circle but close to rectangle. Thus the perimeter of the grain which was equal to the boundary between α-Bi and ε phases could be more suitable if the phase transition was assumed to begin from the boundary. Thus, the boundary indicator γ which was determined from perimeter length l and area of α-Bi S by following equation were compared with each condition.

(6) γ=l/S 

**Figure 10:**
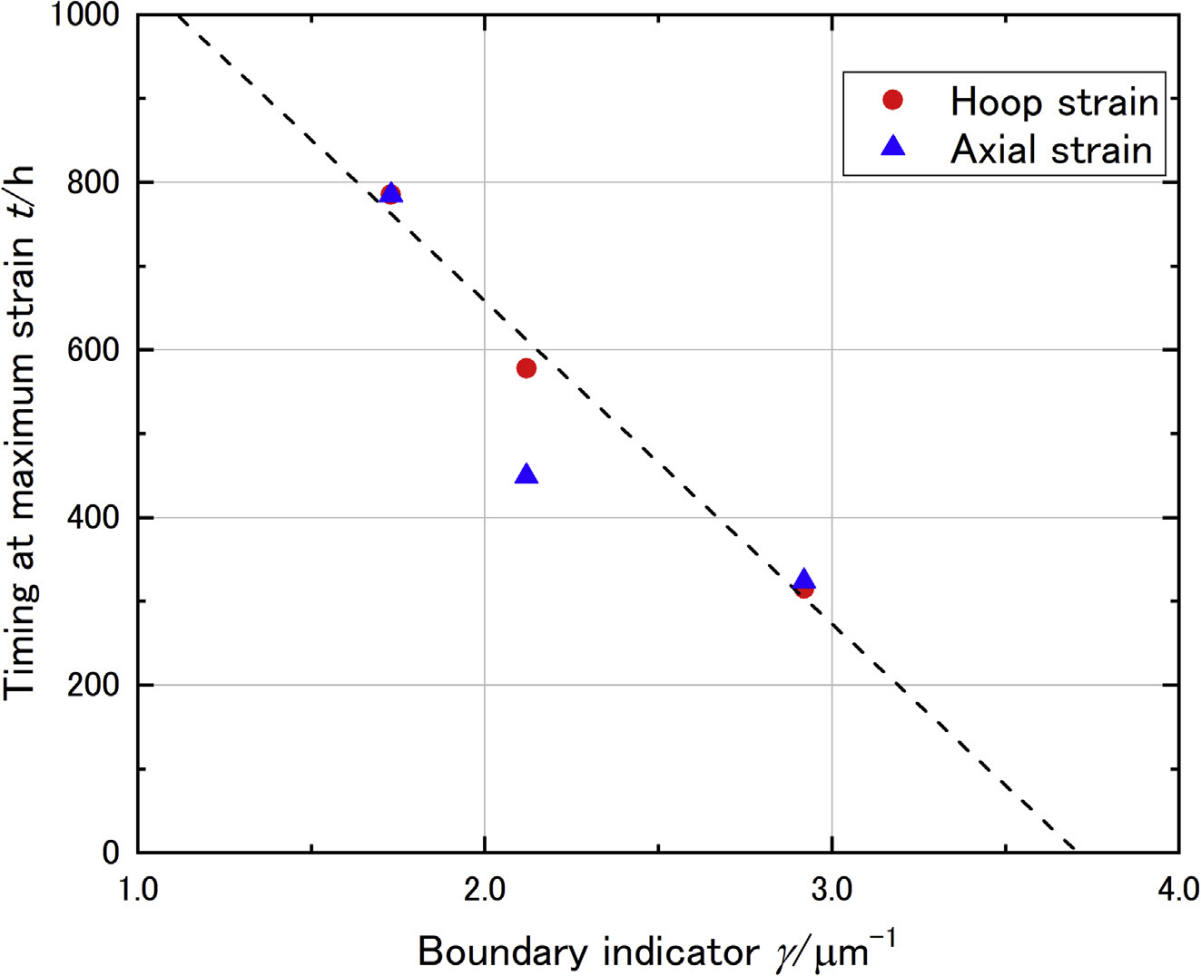
Relation between perimeter and maximum strain reached time which indicated the phase transition might proceed from grain boundary.

Comparison between the time to reach maximum strain and the boundary indicator showed a clear linear relationship (see Figure 10). One data point of axial strain showed relatively shorter time to reach maximum strain, however, the time to reach maximum hoop strain was emphasized since axial strain probably relied on surface situation. As a result, the results from metallographic observation and strain measurement were correlated by determination of the grain boundary.

## 5 Conclusion

Strain for stainless steel cup produced by LBE expansion was measured with different heat treatment. As a result of the measurement, cooling rate was not effective to reduce the strain although the time to reach maximum strain was obviously changed. On the other hand, keeping at higher temperature (50 °C in this experiment) was remarkably decreased maximum strain. This procedure could be helpful in freeze sealed valve design. Metallographic observation revealed linear relationship among cooling rate, grain size, and the time to reach maximum strain.

## Declarations

### Author contribution statement

Naoya Odaira: Conceived and designed the experiments; Performed the experiments; Analyzed and interpreted the data; Wrote the paper.

Shigeru Saito: Contributed reagents, materials, analysis tools or data.

### Funding statement

This work was supported by the Japan Atomic Energy Agency.

### Competing interest statement

The authors declare no conflict of interest.

### Additional information

No additional information is available for this paper.
